# Image-Based Environmental Monitoring Sensor Application Using an Embedded Wireless Sensor Network

**DOI:** 10.3390/s140915981

**Published:** 2014-08-28

**Authors:** Jeongyeup Paek, John Hicks, Sharon Coe, Ramesh Govindan

**Affiliations:** 1 Department of Computer Information Communication Engineering, Hongik University, Sejong 339-701, Korea; 2 Computer Science Department, University of California, Los Angeles, CA 90095, USA; E-Mail: johnhicks@gmail.com; 3 Biology Department, University of California, Riverside, CA 92521, USA; E-Mail: sharonicoe@gmail.com; 4 Department of Computer Science, University of Southern California, Los Angeles, CA 90089, USA; E-Mail: ramesh@usc.edu

**Keywords:** wireless sensor networks, image sensors, sensor applications, tenet, rcrt, cyclops, environmental monitoring

## Abstract

This article discusses the experiences from the development and deployment of two image-based environmental monitoring sensor applications using an embedded wireless sensor network. Our system uses low-power image sensors and the Tenet general purpose sensing system for tiered embedded wireless sensor networks. It leverages Tenet's built-in support for reliable delivery of high rate sensing data, scalability and its flexible scripting language, which enables mote-side image compression and the ease of deployment. Our first deployment of a pitfall trap monitoring application at the James San Jacinto Mountain Reserve provided us with insights and lessons learned into the deployment of and compression schemes for these embedded wireless imaging systems. Our three month-long deployment of a bird nest monitoring application resulted in over 100,000 images collected from a 19-camera node network deployed over an area of 0.05 square miles, despite highly variable environmental conditions. Our biologists found the on-line, near-real-time access to images to be useful for obtaining data on answering their biological questions.

## 1. Introduction

Avian ecologists study the behavior of birds during the nesting season to answer biological questions that relate to the laying, incubation and hatching of eggs and the fledging and survival of nestlings. Studies on the breeding biology of species of birds that normally build their nests in natural holes in trees are part of the research effort that has been taking place for several years at the James San Jacinto Mountains Reserve [1]. For such species, researchers often place wooden boxes, called nestboxes, on trees that birds can use for nesting, because they allow the researcher to view the contents of the nest more readily compared to a cavity within a tree. Nestboxes have been placed around the reserve and have been occupied by breeding birds. However, observing the day-to-day changes of breeding behavior in these boxes is extremely labor intensive. To answer certain kinds of scientific questions, the interior of each box must be checked daily by a biologist in the field or it must be wired with a permanent camera for remote observation. Thirteen of the boxes at the Reserve have indeed been wired to give high quality and high resolution images, but they have limitations. The number of wired boxes is low, as they are restricted to locations near sources of power and Ethernet. Furthermore, in biological studies using nestboxes, it can be hard to predict which nestboxes will be occupied by birds in each year prior to the initiation of the bird nesting season; therefore, a system that relies on wired connections to nestboxes has the disadvantage of being more difficult to relocate when an individual nestbox that has a buried wired connection is found to be unoccupied in a given year. Similar challenges exist for studying the biology of lizards and amphibians using pitfall trap arrays at James Reserve.

To address these issues, we needed an imaging system that can be deployed easily and moved flexibly over a large area at places where wired infrastructures are less available. The system should support a large number of cameras and should operate for long periods of time without excessive maintenance. Furthermore, it needs to be easy to run and re-configure.

In this article, we devised and implemented a low-power, scalable, wireless imaging system using off-the-shelf hardware and Tenet, a readily-available open-source software package for programming tiered wireless sensor networks [2]. We also discuss two real-world deployments of image-based environmental monitoring sensor application using this wireless sensor network system; one for pitfall trap monitoring and another for bird nest monitoring, both deployed at James Reserve.

Low-power sensor networks are not a new theme, and there already exist various systems that can take environmental measurements and return them in near real time. The work most close to ours is [3], which reports a similar deployment for the bird nests at James Reserve, but does not provide a fully end-to-end system, nor does it provide multihop capability, reliability, congestion control and many other features explained in the following sections. Othman *et al.* [4] reported a complete end-to-end wireless sensor network system for swift bird farm monitoring using a similar mote platform to ours, but their sensors were mainly temperature and humidity and did not include a camera for image-based monitoring. Lloret *et al.* [5] presented a WLAN-based camera sensor network for vineyard monitoring, and Hwang *et al.* [6] studied an agricultural environment monitoring server system using a wireless sensor network. Furthermore, Szewczyk *et al.* [7] analyzed a large-scale habitat monitoring application using wireless sensor platforms similar to ours, and Cerpa *et al.* [8] discussed a communication and data aggregation architecture for WSN-based habitat monitoring; however, neither used camera sensors.

However, there are few such systems for low-power high data rate retrieval of images, which are also configurable and usable by those with little knowledge of embedded programming. The large size and inherent complexity of images along with a large number of cameras over a wide area gives rise to routing, reliability, congestion control, power and other issues not seen in lower data rate systems. One known solution stems from a similar deployment for the bird nests at James Reserve [3]. Our deployment uses the same hardware, including the same nestboxes and their locations, but implements a completely revamped software system. We chose the open-source Tenet package, which gives a number of usability and flexibility advantages, as explained in the following sections. The main contribution of this article is in the development and real-world deployment of an end-to-end system that satisfied the requirements of our biologist and the experiences from it.

## 2. Motivation

This section describes the motivations behind our deployments of two wireless imaging applications at James Reserve.

### 2.1. Pitfall Trap Monitoring

Pitfall trap arrays at James Reserve are used by biologists to sample the local population of lizards and amphibians. Biologists deploy arrays of traps in clusters, and when a lizard is caught in a trap, it is tagged and freed. Over time, the count of trapped lizards can be used to estimate the overall population of lizards. To avoid the mortality of lizards and amphibians or any incidentally-captured animals (e.g., small mammals) from a variety of causes while in the trap, the pitfall traps are visited by biologists on a regular basis (on the order of hours) to remove captured animals. Often, however, the visits results in the biologist finding the trap empty. When traps are deployed over a large area (e.g., several square kilometers), the checking of traps represents a considerable investment of time. A wireless imaging system based on a wireless sensor network using low-power cameras can reduce the amount of time that a biologist needs to spend checking traps. Using a low-power wireless imaging system, the biologists can visually inspect images from every trap remotely and only visit a trap when an animal is caught, saving their time and effort.

### 2.2. Bird Nest Monitoring

Avian ecologists study the behavior of birds during the nesting season to answer biological questions that relate to the laying, incubation and hatching of eggs and the fledging and survival of nestlings, primarily during the breeding season, which can last up to three months during spring. Usually, a minimum of 30 nests of a single species of bird is needed to provide a statistically robust analysis (to achieve this, a researcher studying birds in nestboxes would typically place twice that number of nestboxes in the field to give ample “choice” to birds in selecting nest sites, with the hope of achieving 50% occupancy). Each of these nestboxes needs to be checked manually on a regular basis to determine which are occupied by birds and then monitored regularly (in some cases, daily) to take data on the breeding behavior of interest. Such efforts can require a considerable investment of time and labor. The low rate of data sampling makes answering many scientific questions infeasible. In addition, manually opening and inspecting the nestboxes to obtain data can create a disturbance that can result in adult birds abandoning their nest.

To this end, the goal of our deployment is to demonstrate a system that can be used by ecologists to continuously observe the interior of nestboxes spread over a large area for the duration of the breeding season with minimal disturbance to the breeding birds. Our work is focused on recording still images inside nestboxes using a wireless camera system to record bird behavior. In addition, we are measuring environmental characteristics of the immediate nesting environment (*i.e.*, inside the nestbox), including temperature, humidity and dew point, as well as near the nesting environment (*i.e.*, outside of the nestbox). The environmental data and associated nestbox images are being used to answer questions about bird breeding behavior and breeding success. The questions fall into the following areas:

- Nestbox use during the non-breeding season;
- Environmental variation among nestboxes with respect to nest site selection and adult breeding behavior;
- Inter-species competition for nestboxes;
- Laying patterns and behavior;
- Incubation and hatching (pattern/time, asynchrony);
- Fledging (variation among nestlings within a nest).

Biologists can gain insights into the biology of the birds based on the data that have been collected through our system, and this data can help answer significant biological questions, as discussed below.

## 3. Requirements

There are several requirements for our system. First, the system should not miss important events. This means that images should be taken at a relatively high rate, high enough to detect events, such as trapped animals or bird presence/absence, with high accuracy. Second, the system must be scalable enough to monitor a sufficient number of traps and nests to derive statistically robust results. This means that the system should be capable of acquiring and managing images from a large number of sensor nodes spread over a large area. Third is the ease of use by non-computer scientists (e.g., biologists), both in set-up and the operation and maintenance of the deployment, with minimal dependence on computer programmers. The system should allow the user to easily start, stop, modify and reconfigure the application. Furthermore, we need an end-to-end system to allow the operator to monitor incoming images and data in real time. This will allow the personnel who are maintaining the system to detect problems as they happen and to alter various settings to maximize the data return.

In addition to the above requirements, the incremental cost of adding more nodes to the system should not be too high. It should be possible to place sensor nodes at places where direct radio communication to the server is not possible. Furthermore, each camera must be low power to reduce maintenance costs or, with the help of solar panels, eliminate those costs during the monitoring period. All of these requirements have implications for multihop communication, packet reliability, congestion control, mote stability, power consumption, and image processing algorithms.

## 4. System Architecture

This system uses low-power image-sensors (Cyclops cameras [9]) on a tiered wireless sensor network system called Tenet. The Tenet system includes all necessities for basic wireless sensor network programming: drivers, routing protocol, flow control, end-to-end reliability, a two-tier network hierarchy and a simple scripting language for easy programming of applications. These properties, in addition to Tenet's extensibility, make Tenet an ideal choice for our deployments.

Tenet collects images and environmental data from every sensor node and stores them on the local server at James Reserve. Tenet applications run on a local server, and one or more Stargate(s) act as Tenet-masters, which relay commands to and data from the Cyclops nodes. A back-end server retrieves the data from the local server via the Internet and archives and processes the data. These components together give a complete, real-time, end-to-end system. These factors together enabled us to reach our goal of increasing the temporal resolution of data, providing near real-time monitoring, which saved time and labor for the biologist, allowing a larger spatial area to be covered with sensors.

### 4.1. Tenet Advantages

Our wireless imaging system has several requirements, as described in Section 6.3. These considerations have led us to adopt Tenet for our system, as Tenet already addresses most of the above requirements and allows the easy addition of new functionality.

Tenet is a software package for flexibly programming a tiered network of sensors. The Tenet system consists of motes and less-constrained 32-bit platforms, called masters. All applications run on the masters and task motes using a generic tasking API that allows the user to run simple programs on the master nodes to configure, control, sample and process data without having to reprogram the motes. Tenet constructs seamless multi-hop routing over a tiered network of motes and masters, which enables flexible deployment of sensors over a large area. Tenet also provides end-to-end reliable delivery of packets with a built-in congestion control capability. Reliable delivery is an application requirement for our system; otherwise, image quality can be severely compromised. It also allows our system to use loss-intolerant image compression techniques to increase effective network capacity, since these techniques require 100% packet delivery for correct decompression. Congestion control allows our application to adapt its image transfer rate to the network scale and a wireless environment. By using Tenet, we can reuse all of the above networking and sensor data extraction code, thereby significantly reducing application development time.

### 4.2. Software Development

The goal of our applications is to repeatedly collect from every node a Cyclops image along with MDA300 sensor readings as frequently as possible. Developing our imaging applications using Tenet involved adding several new software pieces into Tenet. We ported device drivers for the Cyclops camera and the MDA300 data acquisition board into Tenet, added Tenet tasklets and its APIs for accessing these devices and implemented basic image compression algorithms (described in Section 5) to reduce the image transfer latency. All of these were fairly straightforward to implement in Tenet. Finally, two new Tenet applications, one for the pitfall trap monitoring and another for the bird nest monitoring, were written in the Tenet scripting language [2], as we will describe in Sections 6 and 7.

### 4.3. Back-End Server

The back-end database and image viewer completes our system. We used a database server at CENS (Center for Embedded Networked Sensing), which stores environmental data and images. As mentioned, the images and environmental data are collected at a Linux server running at James Reserve. The data were pulled from this machine and entered into the database at regular, 15-min intervals. The images and data are finally displayed on one of two flash viewers. One allows easy image browsing, while the other is used to monitor the status of the system in real time.

## 5. Image Compression

Our sensor nodes are embedded devices running on battery power. Thus, we needed to develop algorithms that take into account the limited computational and memory resources available on these devices, their limited radio bandwidth and the power constraints under which these devices operate. Image compression will allow us to reduce the number of packets per image to be transmitted over the radio, which will reduce the overall power consumption and allow us to transmit more images for higher resolution data [10]. However, the image compression algorithm must be simple enough to be implemented on these resource-constrained, embedded devices. Specifically, our nodes have 64 kB of SRAM memory, out of which around 5 kB were used for infrastructure code, leaving us with only around 59 kB for image storage and processing. Each 128 × 128 grey-scale image consumes 16 kB of memory, which allows us to have at most three images in the memory for simple processing, like ‘C = PROCESS(A, B)’. Each 200 × 200 grey-scale image consumes 40 kB, giving us no extra room for another image, other than the one that is being taken from the camera sensor. For these reasons, we have employed two different compression algorithms for our pitfall trap deployment and the bird nest deployment.

**Figure 1.**
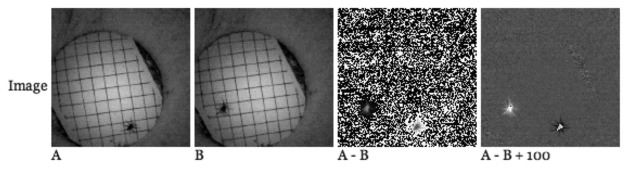
A “background subtraction-based object detection algorithm”example.

In the pitfall trap deployment, the requirement was that we need to ‘detect’ an event when an animal is captured in the trap. If there are no events, biologist need not visit those traps for investigation and rescue. Thus, images are not required to be transmitted when there are no events, but must be transmitted when there are. Once the animal has been rescued, the trap can go back to the no event state. For this reason, we used a simple “background subtraction-based object detection” [11] algorithm on 128 × 128 grey-scale images for the pitfall trap deployment. This seemed to be an ideal choice, not only because of its low memory and computation requirements, but also due to the fact that the cameras are pointed at an enclosed, unmoving background. In this algorithm, if the newly-taken image differs from the previous one, we say ‘detected’. By ‘differ’ we mean, for the pixel with the largest gray scale value difference greater than a threshold value ‘THRESH’, ‘M’ pixels out of ‘N’ pixels around that pixel have a gray scale value difference greater than ‘THRESH’. If ‘detected’, we transmit the image, otherwise not. A threshold value was used to take into account minor differences in the light intensity. Figure 1 depicts an example of this procedure. A newly taken image ‘A’ is compared with the previous image ‘B’, and the background subtracted ‘A-B’ is used for object detection. The black and white dots in the ‘A-B’ image are due to minor (±1) differences in light intensity, which translated into 0/255 gray scale values. Offsetting the ‘A-B’ image with a fixed constant ‘X = 100’ (‘A-B + X’) proves this, where ‘X’ can be any positive constant large enough to mask the minor (±1∼2) differences and noise in light intensity (e.g., X < 4). (In other words, using any value greater than four will result in identical detection algorithm behavior. One hundred was randomly chosen among the values that show the (‘A-B + X’) difffigure nicely for presentation.)

SCOPES [12] also used a similar technique with the background updated using EWMA. However, we found that updating the background using EWMA created an artificial background that does not represent any real background at any instance in time and that created more false positives in our scenario. As we will describe in more detail later in Section 6, our background subtraction-based object detection algorithm not only detected true events, but also had several false positives, motivating improvements. Many of the transferred images were triggered as a result of changes in the intensity of sunlight or when the image was completely dark at night.

In the bird nest deployment, the requirements were different from the pitfall trap deployment. First of all, background subtraction would not be useful, not only because of the sunlight changes, but also because the birds fly in and out frequently. Second, the biologist requested for higher resolution images (200 × 200) than 128 × 128 for their purposes, leaving us no memory space for running any kind of algorithm that required additional memory. Thus, we needed to develop a new image compression algorithm for our bird nest deployment.

**Figure 2.**
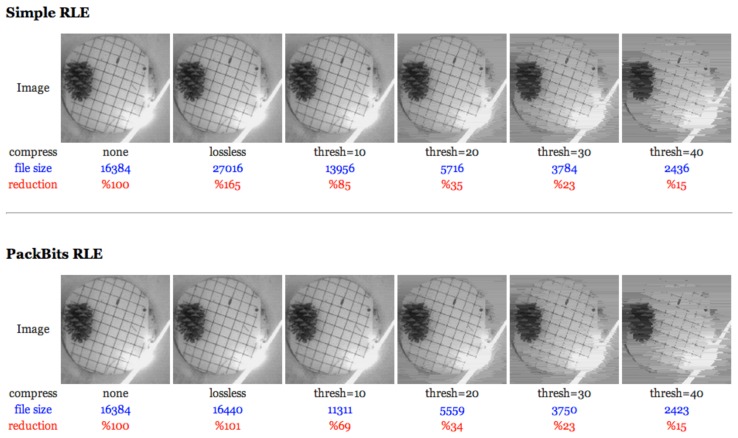
Lossy version of the “simple run-length encoding” (RLE) and “PackBits” algorithms with a threshold example.

For this reason, we have investigated two types of run-length encoding (RLE) on the Cyclops. One was the simple run-length encoding, where everything is encoded in to [length][byte], and another was the ‘PackBits’ algorithm. PackBits is a fast, simple loss-less compression scheme for run-length encoding of data originally developed by Apple. These schemes were chosen because the encoding operation at the mote-end is simple and can be done on-the-fly without requiring a full image size buffer. We modified these two algorithms into lossy algorithms, so that a sequence of values within a threshold range consists of a ‘run’ (in our image data, each byte represents a gray scale value ranging between zero and 255). In other words, a ‘run’ consists of values where the difference between the minimum and the maximum values within that run are less than the threshold. For example, if the threshold is 10 and the original image data are {1, 2, 1, 3, 2, 3, 2, 2, 15, 20, 15, 20, 100, 110, 105, 105, 100, 110}, then this image is compressed into {[8][2],[4][18],[6][105]} and will later be decompressed to image data {2, 2, 2, 2, 2, 2, 2, 2, 18, 18, 18, 18, 105, 105, 105, 105, 105, 105}. The threshold value trades-off image quality for increased compression, and setting it to zero is equivalent to using the original loss-less algorithm. Figure 2 depicts examples from our investigation where you can see that we can achieve a significant data reduction if we can tolerate some lossiness in the images after decompressing the lossy-encoded data. In general, PackBits has better compression performance than simple RLE.

**Figure 3.**
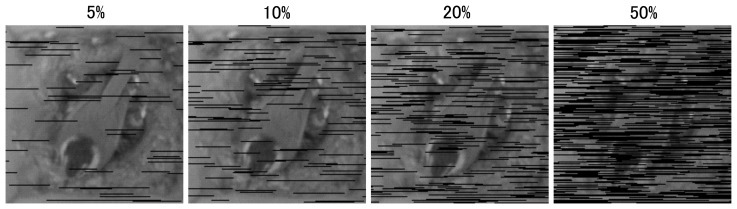
Simple RLE with simulated packet loss of 5%, 10%, 20% and 50%. Average packet loss in our bird nest deployment was around 18%.

One downside of PackBits is that it requires 100% reliable data delivery; if any packet is lost, we cannot decompress it, whereas we can always decompress the simple RLE as long as the fragment size is in even numbers. This is not a problem for us, since Tenet has a reliable transport protocol, but it can cause errors if you are testing it without reliability. One concern for using reliable communication is that it may cause extra retransmissions and, hence, energy consumption. If the images with packet losses are readable and energy overhead is considerable, simple RLE might be a better choice. To validate this, we have conducted an experiment to see the image quality of simple RLE with some packet losses. Figure 3 shows the result of this experiment. The average packet delivery ratio of our deployment was around 82%, with the worst node having 50% packet reception rate (PRR). As you can see from the figure, images with packet losses are not of good quality; our biologist greatly preferred less frequent 100% images over more frequent 80% images. Furthermore, an image with 50% packet loss is not readable. Lastly, the average PRR of 82% translates into ETXof around 1.21, which means that the extra energy consumed by retransmissions are around 21% on average. Our decision was that it is worth spending 21% extra energy to collect 100% images rather than compromising the image quality. Another reason that the biologists wanted high quality images was to perform offline image processing at the backend to automatically detect the presence/absence of birds and also to count the number of eggs from the 100,000+ images that we have collected. This is a separate line of image processing research, which required complete images. Thus, based on the above investigation, we have used the modified PackBits algorithm for the bird nest deployment.

The compression algorithms that we have used in our deployments are simple, yet satisfied the requirements of our users. Other schemes can be considered also. Lee *et al.* [13] explored the energy tradeoffs involved in JPEG compression on energy-constrained platforms, but their findings show that JPEG energy consumption is actually higher on low power platforms due to the longer times needed for these platforms to perform the computation tasks to the desired precision. Table 1 shows the power consumption of our mote platforms and Cyclops camera. TiBS (tiny block-size image coding) [14] and Optimal Zonal 2 × 2 BinDCT [15] are two recently proposed image compression schemes for wireless sensor networks that could be useful for our deployments. TiBS operates on blocks of 2 × 2 pixels and is based on pixel removal. Furthermore, TiBS is combined with a chaotic pixel mixing scheme to reinforce the robustness of image communication against packet losses. However, in our deployments, the primary challenge for implementing these improved algorithms was the practical memory constraint on our motes. The Mica2 and MicaZ motes have only 4 kB of RAM, and the Tenet software, which includes the multihop networking stack, reliable transport protocol and the task library, consumed over 3.6 kB already. However, for example, TiBS requires 22,946 bytes of ROM and 1820 bytes of RAM [14] to implement. There was very little room to implement these sophisticated image processing algorithms on our devices. We agree that newer platforms with more memory can implement better compression algorithms on top of our system. Alternatively, hardware-based image compression, such as the one proposed in [16], can also be a solution if the cost permits.

## 6. Pitfall Trap Deployment

In this section, we discuss a real-world deployment of our wireless imaging system for pitfall trap monitoring deployed at James Reserve. This deployment has its own application and needs, but at the same time, it was in some part an experimental deployment to investigate and gain experience for our next larger-scale bird nest deployment.

### 6.1. Hardware Setup

**Figure 4.**
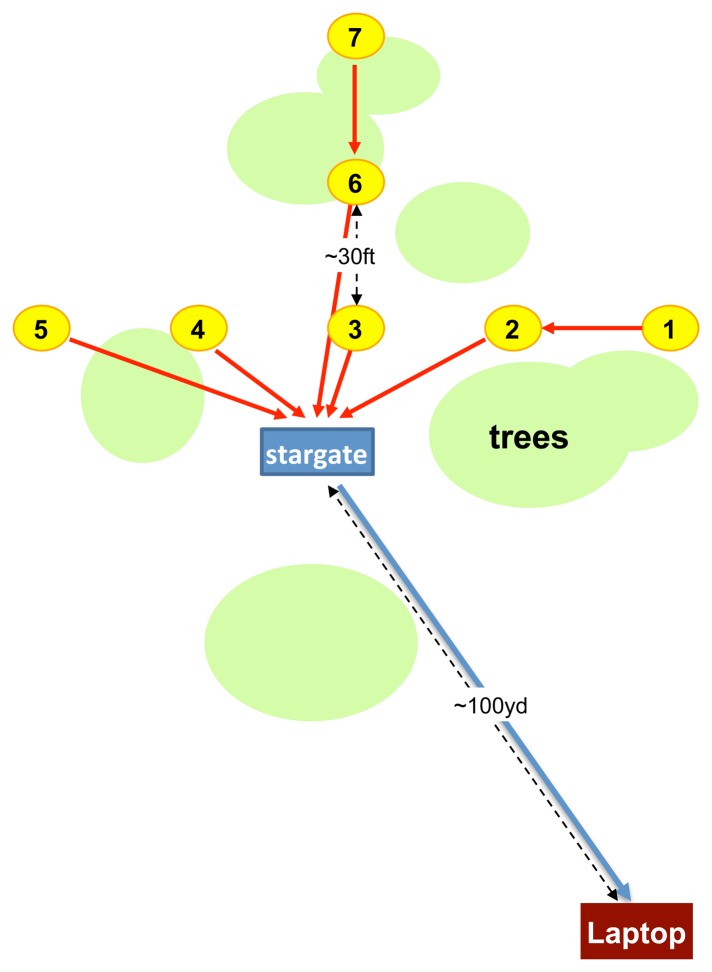
Pitfall trap monitoring deployment topology at James Reserve.

**Figure 5.**
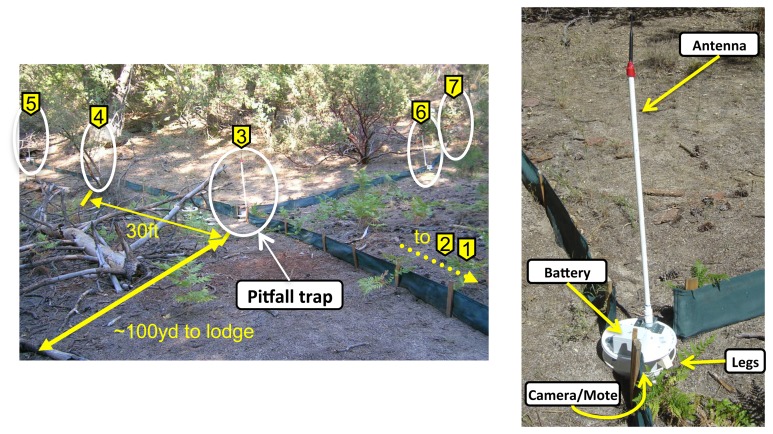
An array of pitfall traps consisting of seven traps in a star configuration **(left)** and the outside of a pitfall trap. Notice the barrier walls on the sides to lead the lizards into the trap and the legs to hold the trap lid above ground. The Cyclops node is under the lid pointing down into the trap **(right).**

Here, we describe the specifics of the hardware and an overview of the configurations used in the pitfall trap deployment. We deployed our system in an array of pitfall traps at James Reserve. Figure 4 depicts the network topology of the deployment. One array of pitfall traps consists of seven traps in a star configuration. We placed seven traps, each equipped with a camera sensor node, one Stargate master near the array and a Linux laptop acting as a server at an indoor location about 100 yards away from the array (Figure 5). The laptop communicates with the Stargate via 802.11, and they together constitute the upper tier of the network. Stargate is connected to a MicaZ mote, which is used to communicate with nearby sensor nodes, which comprise the low power, lower tier of the network. Some of the nodes were behind the trees with foliage, which blocked the line of sight to the base station.

Each pitfall trap contains a pair of D cell alkaline batteries, an antenna and the embedded hardware necessary for imaging and communication. Figure 5(right) shows the outside of a pitfall trap. Plastic traps are all custom-made and contain a sensor node composed of a 2.4-GHz MicaZ mote for communication and a Cyclops camera for imaging. The sensor node was enclosed in a box with a clear plastic bottom through which the camera takes images, while protecting the hardware from dirt and moisture. For most nodes, a pair of D cell batteries was enough to keep them running for more than three days, and all nodes had 9 dBi omnidirectional antennas.

### 6.2. Tenet Application for Pitfall Trap Monitoring

Our goal was to design a triggered data collection system, where each node frequently captures an image, but only sends it to the base station when an animal is likely to be present in the trap. This is necessary to reduce the energy consumption of the battery-powered sensor nodes and to conserve network bandwidth, since each image is 16 kB and data communication is the most significant part of the energy consumption. However, the object detection algorithm we use can exhibit false negatives. If a false negative detection occurs, no image will be delivered at that instant. Even if there actually were an animal in that trap, if it does not move, no image transfer will be triggered at subsequent sampling times, as well. This might result in a missed and eventually dead animal. To avoid this, we designed our application to take an image every 2 min and to transfer the image only if an object is detected, but also to transfer at least one image every 30 min regardless of the detection. Here is a Tenet task that realizes this relatively complicated logic.

Repeat(120000ms)
-> ImageDetect(TAKE_NEW, FLASH_ON, BW, 128, 128, x)
-> Count(y, 0, 1) -> Mod(y, y, ′15′) -> Eq(y, y, ′0′)
-> Or(x, x, y) -> Store(z, x) -> DeleteAllAttributeIf(x)
-> Send(E2E_ACK)
-> Retrieve(z) -> Not(z, z) -> DeleteActiveTaskIf(z)
-> DeleteAttribute(z) -> ImageDetect(RESET)
-> ImageFetch(LAST_IMAGE, 40, BW, 128, 128, out)
-> SendStr();

In this task, ImageDetect() takes a new image and invokes a background-subtraction-based algorithm to detect noticeable differences between the new and previous image. Then, ImageFetch() retrieves the last image stored in the memory. Thus, the preceding task takes an image every 2 min and transfers it only if some change has been detected in the image or every 15th run (every 30 min). If an object is not detected and it is not the 15th run, the task sends a small message (*x* ≡ 0) using Send(E2E ACK), as a keep-alive. Otherwise, it transfers the last image using SendStr() and resets the detection background. Each image is a 128-by-128 grey-scale image, whose size is 16 KB, and each packet can contain up to 40 bytes of image fragment data; so, each image requires 410 packets.

These Image-style tasklets are different from other tasklets in that they are executed on the cameras themselves. Image processing requires memory and MCU cycles beyond the capabilities of the current generation of motes, and the Cyclops board was designed to provide specialized image processing tasks. For this reason, these Image-related operations are implemented within the camera themselves. This design allows any resource-intensive operation to be performed off-mote and can be applied to other specialized external sensors with on-board processing. Finally, the Cyclops was powered off between image captures to save energy. In this case, it remained powered off most of the time and was powered on for only a short duration every 2 min.

### 6.3. Deployment Experience and Results

**Figure 6.**
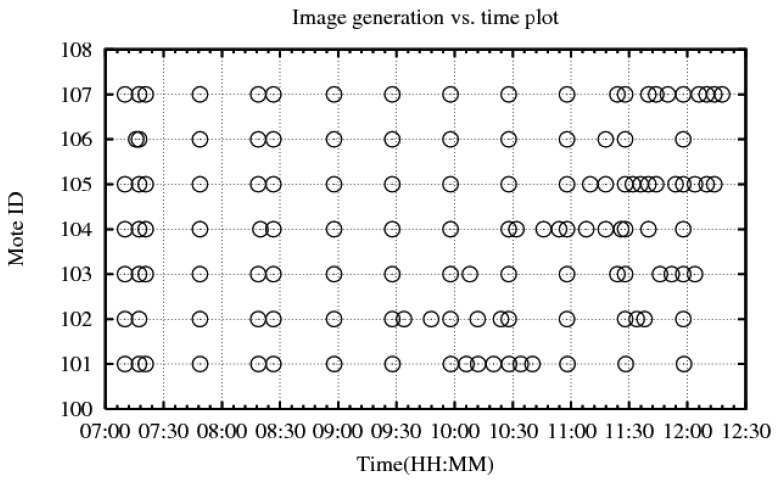
Image-transfer vs. time plot: an image is generated every 30 min, in addition to whenever an object is detected.

**Figure 7.**
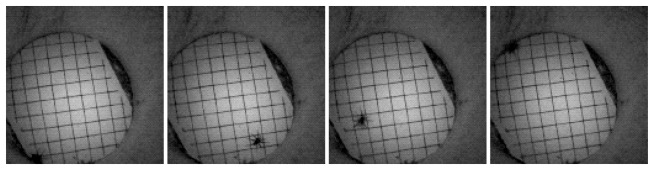
A spider triggered image transfers.

**Figure 8.**
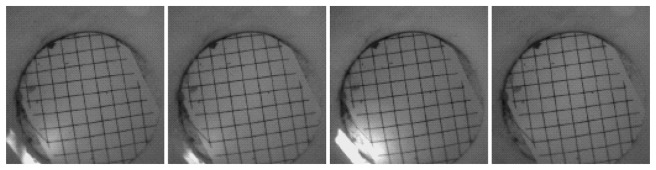
Sun/shade change caused image transfers.

**Figure 9.**
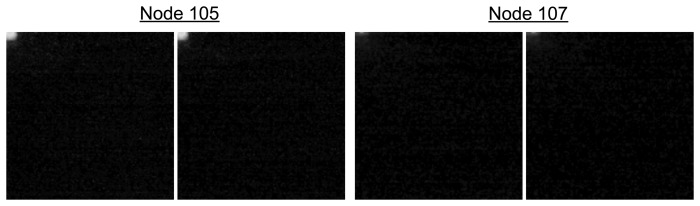
Darkness without IR flash generates continuous false positives.

Our deployment lasted three days, and the network was operational only during the daytime hours (7 a.m.–7 p.m.). We closed the traps during the night, since the biologists were mainly interested in species of animals that are active during the day. Since we had an LED flash equipped on each camera node, our system would have worked for a study at night also if that had been the purpose. We have collected 589 images, out of which 588 were complete; there was one incomplete image from a node that almost ran out of battery. Figure 6 plots the time when each has transferred an image on the last day of the experiment. Unfortunately, we did not capture any animal, probably due to the cold weather. However, there was one time period where image transfer due to object-detection was continuously triggered. This is when we caught a spider (Figure 7). Many of the transferred images were triggered as a result of changes in the intensity of sunlight (Figure 8), and some others were intentionally triggered by us to test the system. Image transfer due to sunlight/shade change is a false positive for our object detection algorithm that we had not anticipated in the lab environment. Furthermore, there were a couple nodes that were continuously transferring images during the night (Figure 9). It turned out that the IR flash was malfunctioning on those nodes, and complete darkness was also generating false positives in our object detection algorithm. For there reasons, we have modified our image compression strategy for our next bird nest monitoring deployment.

The main lessons learned from our pitfall trap deployment are as follows.

- It is feasible and practical to use an image-based wireless sensor network for effective pitfall trap monitoring. Our system is flexible, supports multihop and satisfies many application requirements described in Section.
- However, the background subtraction-based object detection algorithm may result in frequent false positives, either due to sunlight intensity changes during the day or complete darkness when not equipped with an IR flash.
- An IEEE 802.15.4-compliant 2.4-GHz radio does not propagate well in mountain environments with dense foliage, and lower frequency radio may be a better choice.

## 7. Bird Nest Deployment

Here, we describe the specifics of our deployment of the bird nest monitoring application at James Reserve, including the overview of the configuration, hardware setup, our application, deployment experiences and the results.

### 7.1. Hardware Setup

**Figure 10.**
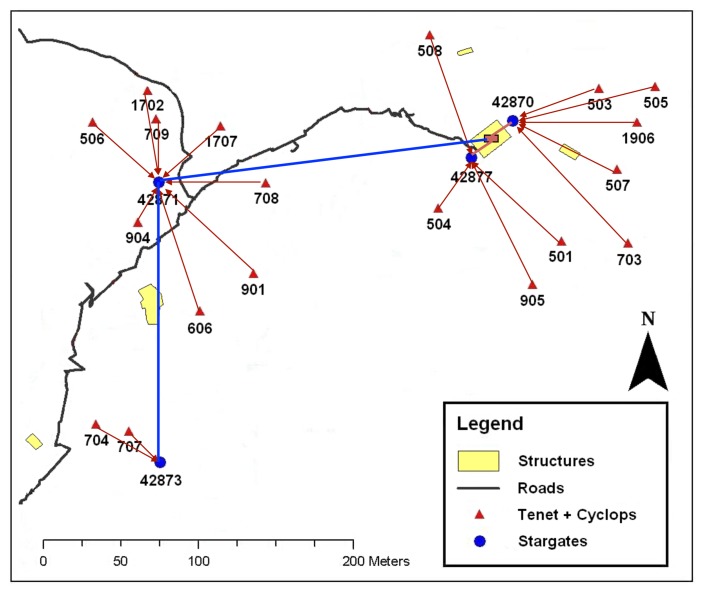
Map of the location and topology of our deployment at James Reserve.

We have one Linux server machine running in the James Reserve server room. The server communicates with four Stargates, which have been placed around James Reserve, and together constitute the upper tier of the network. The server and the Stargates are connected via Ethernet or 802.11. Each Stargate is connected to a Mica2 mote, which all have 8.5 dBi omnidirectional antennas. These Mica2s are used to communicate with nearby Cyclops nodes, which comprise the low power, lower tier of the network. This network topology is detailed in Figure 10. The nestboxes are generally sparse, being spread 50 to 100 m apart in areas of dense trees and foliage. Newer 2.4-GHz radios tend to perform poorly in these environments, since they do not penetrate foliage well. This is the reason why we used Mica2s with 433 MHz radios instead as our wireless communication hardware in the mote-tier. This drastically limits available bandwidth, but propagates farther through the foliage. While our system does support multihop routing (and we did log some temporary multihop paths), nestbox placement was determined from a previous, single-hop deployment, which, when combined with the large Stargate antennas, eliminated most multihop route formations [3].

**Figure 11.**
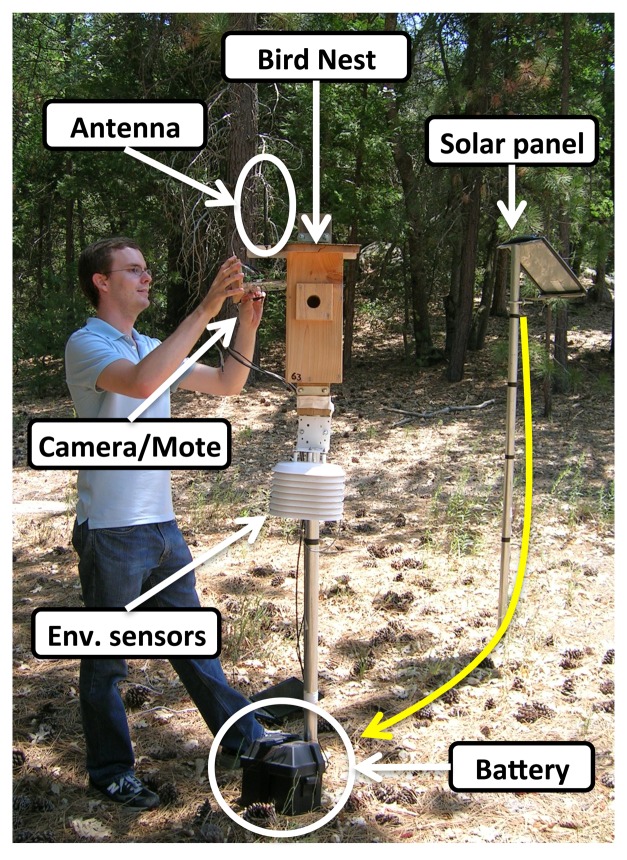
The outside of a typical nestbox. Notice the solar panel mounted at a distance for optimal sunlight, the nestbox mounted on a pole to avoid predation and the shelf at the top holding the hardware.

Each sensor node contains a nestbox, power infrastructure and the embedded hardware necessary for imaging, environmental sensing and communication. Wooden nestboxes are all custom-made and contain a removable shelf in the top of the box, which holds the Mica2 mote for communication, a Cyclops camera for imaging and an MDA300 board for environmental sensing (internal temperature, internal humidity, external temperature, external humidity and voltage sensors are connected to the MDA300 board). Figure 11 shows the outside of a nestbox. The shelf has a clear plastic bottom through which the camera takes images, while protecting the hardware from dirt and disturbance by nesting birds. The camera was facing downward toward the bottom of the box. The power infrastructure is a medium-sized 12-V sealed lead acid battery continually charged by a solar panel. For most nodes, the solar panel provides enough power for unlimited node uptime (on a few nodes, the solar panel did not receive enough solar radiation throughout the day, which resulted in failed nodes due to the loss of power.). Finally, while most nodes had small 433-MHz dongle antennas mounted on the top, a few nodes with extremely poor connectivity required 9 dBi directional antennas pointed at the nearest Stargate.

### 7.2. Tenet Application for Bird Nest Monitoring

The goal of our application is to repeatedly collect from every sensor node a Cyclops image along with MDA300 sensor readings as frequently as possible. To achieve this, the following two Tenet tasks were used;

TASK1:
ImageGetPackBits(0, 40, 100, 1, 16, 200, 200, 5, x) -> SendRcrt();
TASK2:
Wait (1000millisec)
-> MDA300(ch0, ADC1, a, 0) -> MDA300(ch0, ADC2, b, 0)
-> MDA300(ch0, ADC3, c, 0) -> MDA300(ch0, ADC0, d, 153)
-> MDA300(ch1, ADC0, e, 153) -> Send(E2E_ACK);

The first task takes a 200 × 200 resolution grey-scale image, compresses the image using modified PackBits algorithm with a threshold of five, fragments it into 40 data bytes per packet and sends the packets back using the RCRTprotocol [18]. The second task reads the five ADC channels on the MDA300 board and sends the data back using the packet reliable transport protocol. Then, a simple server-side script executes the first task, waits for all responses to arrive, executes the second task and repeats this process indefinitely.

In this way, we are practically running the imager as soon as the image transfers for the previous image transfer task have completed for the whole network. Due to the time-varying link and network conditions, individual image transfer completion time will differ between nodes. Therefore, the server side application checks whether all nodes have competed by checking whether there are any partially decoded images and waits until all started image transfers have completed. Once complete, the application re-issues the next task. It does not wait for an image transfer that has not started at all (e.g., down node). Any partially-completed transfer will timeout after some time (e.g., 2 min without any packet reception).

For compression, we have used the modified PackBits algorithm, as described in Section 5. We chose a threshold of ‘5’ as a reasonable balance; we achieved a 16.7% reduction in image size, while experiencing little visible degradation in image quality.

The RCRT protocol [18] performed congestion control and rate adaptation, as well as loss recovery during the image transfers. Reliable delivery was critical, since the PackBits algorithm requires 100% packet reception for successful decompression. Congestion control was required, since a large number of nodes were sending images simultaneously, and rate adaptation allowed the system to dynamically adapt to the network condition and deliver images as fast as possible. In our deployment, each node delivered an image approximately every 8∼15 min depending on the network and environmental conditions. During the three-month period, RCRT successfully delivered 99% of the images (achieved 100% packet delivery per image for 99% of the images) while transporting approximately 83 million packets in total.

Finally, note that there was a similar deployment at James Reserve before [3] that did not use Tenet. Our deployment not only replaced the previous system, but also improved upon that in several aspects: multihop communication, reliable data delivery, easier-to-use end-to-end system, etc.

### 7.3. System Evaluation

To evaluate our system, we first discuss deployment experiences and then give an overview of measured system metrics from two perspectives. We measure sensor node uptime and occupancy, as well as network behavior.

#### 7.3.1. Deployment Experience

As much of the deployment hardware was already in existence from previous deployments, most of our work in the preparation was spent developing the software using the Tenet system. Our remaining time was spent deploying the hardware with two additional trips to James Reserve for routine system maintenance. We deployed eight nodes during a trial run in early May, then deployed the entire 19-node system on 9 May and brought the system up in the late afternoon. The official end date for our deployment was exactly three months later, 9 August at 5 PM. The server machine at James Reserve is connected to the Internet, and thus, we were able to remotely monitor and reconfigure the deployment. We needed to do this several times when either parameter changes were made to the application or a diagnosis of the network was required. As time went by, there were three distinctive failure events that were observable remotely: (1) unreachable sensor node; (2) sensor node pingable, but not returning images; and (3) rebooted Stargate. The first symptom was usually due to a depleted battery, which, in turn, was usually due to a solar panel being in the shade for an extended amount of time. Broken antenna connectors and malfunctioning power systems were other reasons. The second symptom was either due to low battery (not high enough to activate the Cyclops camera) or a loose connection between the mote and the Cyclops. We believe that one of the Stargates rebooted several times during our deployment, due to power outages(James Reserve power is supplied from generators and solar panels, so there are occasional brownouts and outages). We returned to James Reserve twice, once on 4 June and again on 18 July to fix failed nodes and base stations. All repairs were minor and consisted of replacing improperly functioning 12-V batteries, reconnecting connections that had come loose and adding directional antennas to nodes with poor connectivity. These repairs could, in larger deployments, be taken care of by moderately technically-aware domain experts.

**Figure 12.**
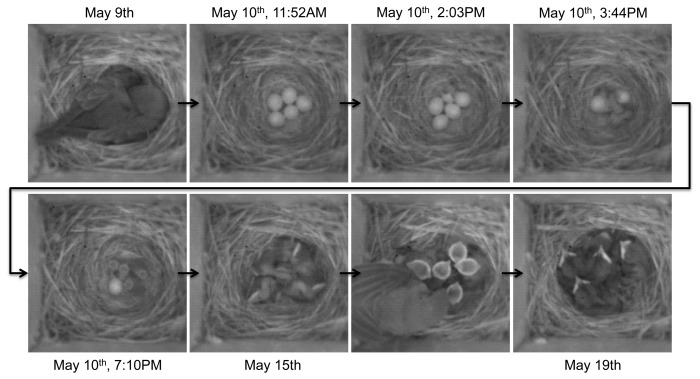
Subset of selected images captured by a camera node during the post-hatching phase. Biologist can detect the occupation of the nest, the type of species, the number of eggs, hatching phases, *etc.*, using these images.

**Figure 13.**
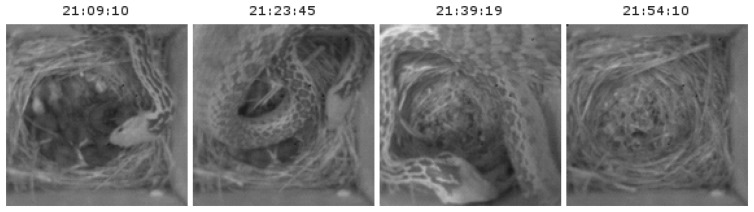
A series of images captured during the post-hatching phase. Notice the snake entering the box and leaving in under an hour, while all of the nestlings have disappeared, an event that would be missed with manual, daily observations.

In total, our system collected 102,173 images from 19 nestboxes, providing biologists with information about nestbox occupancy and the breeding behavior of three species of birds. Figure 12 is one subset of those images captured by a camera node during the post-hatching phase. One highlight of the observations made from the images was that of a snake that was able to enter the nestbox and consume nestlings on 19 May. This unusual event has been captured in a series of images in Figure 13. Without these images, using traditional methods of a biologist manually checking nestbox contents daily or even less frequently, the reason for the disappearance of nestlings from the nest would have been unknown.

#### 7.3.2. Node Examination

**Figure 14.**
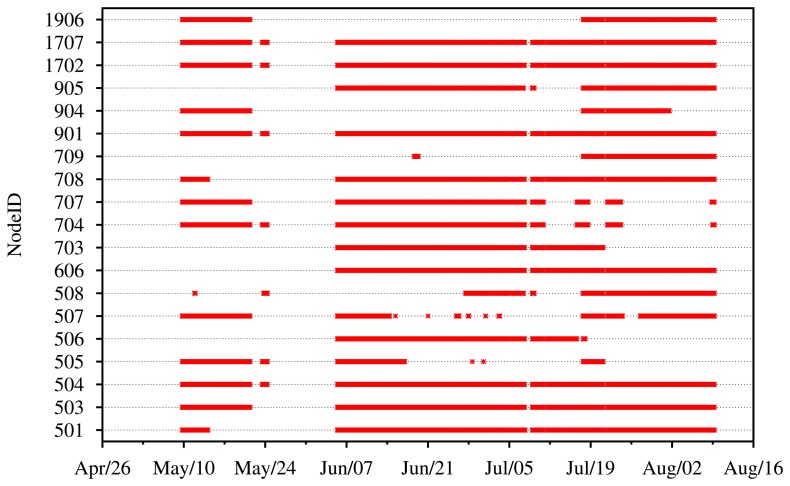
Node uptime of all camera nodes over the three-month deployment period over the season.

Figure 14 shows the uptime of the 19 nodes in our system. There was one major system outage between 21 May and 5 June, which was due to power failures at James Reserve, after which the system did not come up. On 4 June, we returned to the reserve to rectify the issue. On 18 July, we returned a second time to further fix broken nodes. Batteries were replaced and solar panels adjusted on Nodes 1906, 904 and 709. Unfortunately, we almost immediately had a power outage with Stargate 42,873, to which Nodes 704 and 707 connect. Not counting the two-week power outage at James Reserve, our node uptime ranges from about 90% for our stronger nodes, which had no power issues, down to around 40% for our weaker nodes. Node 709 was our weakest node, returning very few images until its battery was finally replaced.

#### 7.3.3. Network Evaluation

**Figure 15.**
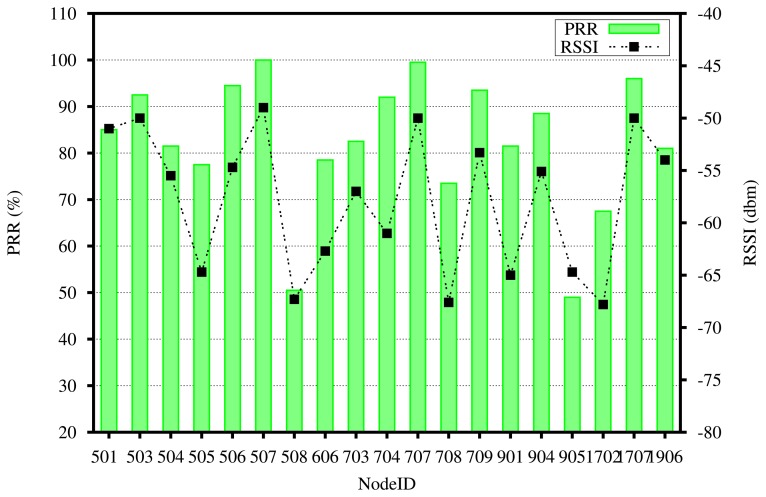
End-to-end packet reception rate (PRR) and received signal strength (RSSI) to the next hop node representing the link connectivity of each node.

As there were several times when the image application was stopped for maintenance or debugging purposes, our networking log files are discontinuous. We present the observations we have made for one week during 21 July ∼28 July. During this period, there were total of 9940 attempts to transfer an image from a total of 19 nodes. Among these attempts, 1489 were made by three nodes when they had low battery and, thus, were unable to turn on the camera. Out of 8453 actual image transfer attempts, 79 attempts resulted in incomplete transfers. Hence, 8372 image transfers, which corresponds to 99% of initiated transfers, were completed during this one-week period. For these complete images, the average data rate achieved by the network was 1.1 packets/s per node, and the average number of packets required to deliver one image was 833.2 (the size of each image is 40 kB, which means 1000 uncompressed packets, but the number of encoded packets differ, since the compression ratio differs for each image), which translates to an average compression ration of 16.7%. As a result, one image transfer took 12.6 min on average. This was achieved despite extremely poor link connectivity (e.g., Node 905 had an end-to-end packet delivery ratio (PRR) of less than 50%). Figure 15 shows the PRR along with their received signal strength (RSSI) readings to the nearest routing parent node for each node during the deployment period.

#### 7.3.4. Lessons Learned

The main lessons learned from our bird nest deployment are as follows.

- It is feasible and practical to use an image-based wireless sensor network for effective bird nest monitoring. Our system is simple to use, scalable, flexible, supports tiered architecture and multihop and satisfies the application requirements described in Section 3. We have captured important biological events, and our biologist users found our system useful for their research purposes.
- However, it is challenging to implement sophisticated image compression algorithms on the resource-constrained sensor platforms. Simple run-length encoding or PackBits algorithms can be effective alternatives, but their compression ratios are limited by the image quality.
- It is possible to use solar panels for providing energy to camera sensors indefinitely without worrying about battery life. However, for long-term deployments such as ours, the growth of foliage and their shade must be considered when placing solar panels for powering the sensor nodes.
- Rate and congestion control is very effective, if not a must, for achieving a high image delivery ratio in a highly dynamic network environment.

## 8. Conclusions

We have developed, deployed and demonstrated a complete, scalable, end-to-end image-based sensor application for environmental monitoring to unobtrusively observe biological phenomena. Our deployment has shown that our system design has met most of the application requirements: sufficient image transport rate for our biological study, scalability exceeding that of wired cameras, ease of use and flexibility of deployment. Tenet software made it easy to monitor, modify and reconfigure the application behavior from a remote location without re-programming the motes. Rate-controlled reliable transport along with the tiered architecture resulted in an image retrieval rate of approximately 3∼4 images per node per hour. Future deployments can further improve this by using a better compression algorithm or newer-generation radios. Finally, our system proved to be scalable. Multihop tiered routing of our sensor network system enabled a larger network with greater spatial reach.

## Figures and Tables

**Table 1.** Power consumption of Mica2/MicaZ [17] and Cyclops [9].

**Operation**	***Mica2***	***MicaZ***
Idle	9.6 mW	9.6mW
MCU Active	24.0 mW	24.0mW
MCU + Radio TX (0 dBm)	76.2 mW	63.0 mW
MCU + Radio RX	45.3 mW	69.9 mW

**Operation**	***Cyclops***	

Capture Image	42 mW	
Extended Memory Access	W = 53, R = 50 mW	
MCU Active	28 mW	
